# Promoting Virtue or Punishing Fraud: Mapping Contrasts in the Language of ‘Scientific Integrity’

**DOI:** 10.1007/s11948-016-9858-y

**Published:** 2016-12-19

**Authors:** S. P. J. M. Horbach, W. Halffman

**Affiliations:** Faculty of Science, Institute for Science, Innovation and Society, Radboud University Nijmegen, P.O. box 9010, 6500 GL Nijmegen, The Netherlands

**Keywords:** Scientific integrity, Scientific misconduct, Scientometrics, Co-word analysis, Discourse, Research integrity

## Abstract

Even though integrity is widely considered to be an essential aspect of research, there is an ongoing debate on what actually constitutes research integrity. The understanding of integrity ranges from the minimal, only considering falsification, fabrication and plagiarism, to the maximum, blending into science ethics. Underneath these obvious contrasts, there are more subtle differences that are not as immediately evident. The debate about integrity is usually presented as a single, universal discussion, with shared concerns for researchers, policymakers and ‘the public’. In this article, we show that it is not. There are substantial differences between the language of research integrity in the scientific arena and in the public domain. Notably, scientists and policymakers adopt different approaches to research integrity. Scientists tend to present integrity as a virtue that must be kindled, while policy documents and newspapers stress norm enforcement. Rather than performing a conceptual analysis through philosophical reasoning and discussion, we aimed to clarify the discourse of ‘scientific integrity’ by studying its usage in written documents. To this end, large numbers of scientific publications, policy documents and newspaper articles were analysed by means of scientometric and content analysis techniques. The texts were analysed on their usage of the term ‘integrity’ and of frequently co-occurring terms and concepts. A comparison was made between the usage in the various media, as well as between different periods in which they were published through co-word analysis, mapping co-occurrence networks of significant terms and themes.

**Electronic supplementary material:**

The online version of this article (doi:10.1007/s11948-016-9858-y) contains supplementary material, which is available to authorized users.

## Introduction: The Meanings of Integrity

In recent years, an increasing number of commentators have expressed concerns over ‘research integrity’ or ‘scientific misconduct’. They typically point out that integrity is an essential aspect of research, of great importance to both scientists and their stakeholders (Godecharle et al. 2014). There seem to be good grounds for such concerns: a succession of scandals has challenged the effectiveness of the peer-review system as a means of preventing misconduct in science (e.g. Abma 2013; Consoli 2006; van der Heyden et al. 2009), and surveys indicate disconcerting levels of misconduct and ‘questionable research practices’ (Fanelli 2009; John et al. 2012). Research misconduct and integrity have made the headlines of academic publications, with journal editors and research leaders ringing the alarm over the spread of misconduct and the emergence of novel forms (Martin 2013; Bohannon 2013). Mass media have picked up the issue and policy attention is on the rise, with more and more policy programs aiming to ‘improve research integrity’ (e.g. KNAW 2014). In the wake of this attention, there is now a growing body of research on scientific integrity and misconduct (e.g. Fanelli 2013; Hiney 2015; OECD 2010; Steneck 2006).

In this article, we want to take some distance from these concerns and investigate the language that is being used to express them, instead of trying to define what research integrity is, or trying to measure the occurrence of misconduct, which are the predominant concerns in current research (e.g. Bretag and Carapiet 2007; Fanelli 2009; John et al. 2012; Komić et al. 2015; Martin 2013; Martinson et al. 2005; Salwen 2015; Science Europe 2015; Steneck 2006; Stroebe et al. 2012). We have several reasons to be puzzled by the universalizing, essentialist language of ‘integrity’. Interestingly, in spite of a seemingly widely shared concern, there is an ongoing debate over what actually constitutes research integrity. The understanding of integrity ranges from the minimal, which only considers falsification, fabrication and plagiarism (FFP), via a range of questionable research practices (QRP), to the maximum, which blends integrity into science ethics (Steneck 2006). However, underlying this obvious range, there are more subtle differences that are not as immediately evident. There are diverging notions of integrity as an individual or as an institutional responsibility, or of integrity as adherence to a clear set of norms versus an aspiration to an unobtainable ideal. The claim that integrity is essential to science, seems to sit uneasily with such a divergence in its understanding.

The absence of a clear and commonly held understanding of integrity in research is sometimes believed to hamper the promotion of scientific integrity and the prevention of misconduct. Hence several authors and policy initiatives have called for more uniform definitions (Hiney 2015). However, the meaning of the word ‘integrity’ is shaped by the demands of disciplines and the political and cultural context of individuals who pursue an academic investigation (PRINTEGER 2015). The diversity in the meaning of ‘scientific integrity’ may well be constitutive of the debate, reflecting the diversity of research practices, rather than a temporary confusion over definitions.

Similarly, the discourse of scientific integrity has varied considerably over time. Even though integrity is currently considered to be crucial to the scientific enterprise, science seems to have gone through several golden ages without expressing such concerns for integrity, despite the fact that scientific misconduct has always existed. Concerns of the past have ranged from ardent but illusory observations such as the notorious N-rays (Nye 1980); dubious claims over the priority of discovery as with Mendel’s laws (Magner 2002); the ethically questionable and methodologically shoddy research of eugenics (Kevles 1985); or even the appropriate conditions of human dissection for Galen in Antiquity (Magner 2002). Currently, there are indications that some forms of misconduct are on the rise and that new threats to integrity have appeared, such as predatory journals or the abuse of scientometric indicators (Martin 2013). However, even though the term ‘scientific integrity’ is a relatively recent expression, concerns over misconduct and the appropriateness of certain scientific practices certainly have been around for a long time and with some considerable thematic variations (Resnik 2003).

In most of the current research on the topic, the debate about integrity is presented as a single, universal discussion, outside of history and with shared concerns for researchers, policymakers and ‘the public’. In this article, we show that there is not one shared debate over ‘research integrity’ not even in current times. There are substantial differences between the language on research integrity in the scientific arena and in the public domain. Notably, scientists and policymakers adopt different approaches in defining and discussing integrity in research and their approaches to achieve ‘integrity’ seem to be growing further apart. We argue that this growing divergence in the discourses on research integrity is of concern for policymakers and researchers alike. It is particularly doubtful whether a divergence in approach between these parties is desirable. Indeed, major discrepancies between the focus of those setting and formulating the norms and those having to live up to them can hardly be considered advisable.

With an analysis of the *language* of ‘integrity’ and ‘misconduct’, we work in the tradition of Science and Technology Studies that questioned the older essential approaches such as the Mertonian sociology of science (Merton 1973; Storer 1966). Social scientists such as Tom Gieryn or Michael Mulkay pointed out how essentialists understandings of what science *is*, are articulated in specific circumstances in quite different ways. This highlights the arguments and negotiations that are involved in articulating what is proper science, what forms of knowledge can rightfully claim the cognitive authority of science, and what the consequences of such claims are (Gilbert and Mulkay 1984; Gieryn 1983, 1995; Mulkay et al. 1983) While some of the reflexive studies of language have led to relativism (Ashmore et al. 1995), and an inability to produce normative guidance (Radder 1992, 2010), such is not our goal. Rather, by analysing the language that misconduct and integrity are expressed in, we want to point to assumptions and patterns, making them explicit for reflection and improvement. This follows the use of discursive analyses in interpretative policy analysis (Rein and Schön 1993; Yanow 1996; Hajer and Wagenaar 2003; Metze 2007), even though we will use quantitative techniques to do so.

This article presents an empirical analysis of word usage in large amounts of scientific publications, policy documents and newspaper articles, analysed by means of scientometric and content analysis techniques. The texts were analysed on their usage of the term ‘integrity’ and of frequently co-occurring terms and concepts. A comparison was made between the usage of these terms in the various media, as well as between the different temporal periods in which they were published, in order to show contrasts in the scientific and public discourse of integrity, as well as changes over time. The section “Theoretical Framework: The Dimensions of Integrity” will present a theoretical framework of the dimensions of the concept ‘integrity’. Several definitions will be discussed, as well as the basis on which these definitions differ. This will inform the empirical analyses of our data. The “Methods and Data” section describes the employed research methods and the data studied. The results of the empirical study will be presented in “Results: Discursive Patterns”. These results will be split in three sections: first, the contemporary differences between the language of integrity in the scientific and public debate will be presented. Second, the evolution of the concept of integrity as well as the genealogy of the term will be discussed. Last, differences in the policy debate in various countries will be presented. The final section summarises the conclusions of our study and considers its implications for the policy debate on scientific integrity.

## Theoretical Framework: The Dimensions of Integrity

In spite of an often-assumed normative consensus about what constitutes ‘good science’, there are many definitions of integrity and misconduct and they vary substantially. In fact, attempts to define research integrity have been going on for decades, with fundamental disagreements about what should be the focus and the limits of public and professional concern (Steneck 1999, 2006). Even though some of this variation concerns variation in words used for the same concept, there is also a more fundamental disagreement. One source of disagreement stems from what the definitions are for. For example, are definitions of integrity needed to articulate procedures to trace and punish misconduct fairly, or are these definitions also needed to inspire scientists to higher moral standards? Another source of disagreement stems from differing assessments of whether research communities are able to maintain and even police their standards, or whether some form of closer public scrutiny is required (Resnik 2003; Fanelli 2011). Hence, a difference in understanding of what constitutes ‘integrity’ reflects differing notions of what constitutes ‘good science’, of how this ideal should be achieved, and of how responsibilities for these issues should be shared between researchers, research institutes, research communities, and public institutions (Guston 1999). Based on a (brief) review of the literature on scientific integrity and misconduct, we can identify three contested issues that are central to the debate on how integrity is to be understood: broadness and intentionality, value-based versus norm-based, and the relevant components of the research process. We will then use these three issues as discursive dimensions in the empirical analysis.

### Broadness

According to Fanelli, differences in the definition of integrity and misconduct take place along two main lines of contention: broadness and the closely related issue of level of intentionality. Along the line of broadness, one can distinguish between the very narrow definitions of misconduct, limiting it to falsification, fabrication and plagiarism (FFP); the broader definitions, including what is currently referred to as questionable research practices; and the conceptually open definitions, including unethical behaviours not strictly linked to research practices (Fanelli 2011). FFP involves infringements of basic conventions in data handling and in the acknowledgement of sources, mainly relating to the publication system. Some even argue that misconduct should be redefined as ‘distorted reporting’ (Fanelli 2013). As such, FFP is a relatively specific slice of the wider institutions of the sciences. Broader definitions include questionable research practices, such as undue influence of clients over the definition of research questions, hypothesising after results are known, inadequate data management, or even inadequate supervision of PhD students. At the far end of this spectrum, integrity blends with general research ethics. One objection against the broadest definitions is that it becomes unclear what the rules are and hence unclear when scientists have really done something wrong, or even deserve some form of punishment. In a similar vein, in a critique of the broad definition of misconduct adopted by the Swedish Research Council, Salwen (2015) argues that any definition has to satisfy the ‘ordinary language condition’: “It should be consistent with how the terms are used by scientists” (Salwen 2015). His concern is that definitions used by policymakers become too remote from the daily practice of science and hence no longer support research, but become an unwieldy obstruction.

The line of intentionality mainly refers to the definition of misconduct, rather than integrity. Three main categories can be distinguished on this line: intentional acts, performed with the intention to deceive; grossly negligent acts, done in ‘reckless regard for the truth’; and careless acts, which discard ‘the standards of a reasonable, normal person’ (Fanelli 2011). These issues clearly relate to the repercussions of integrity breaches, as intentional acts are associated with more serious levels of culpability, as they are in the legal system. The more serious forms of misconduct, committed with intent to deceive in order to claim career advantages, are generally the ones leading to formal procedures and penalties. Fairness of procedures and clarity about rules are arguments to keep the term ‘misconduct’ reserved for intentional acts and possibly grave forms of neglect, rather than mere sloppiness. For those who are less optimistic about the potential of punishment to achieve research integrity, a restriction to intentional acts sacrifices too large a section of integrity issues in the name of procedure. In this sense too, there is a camp in favour of a narrow definition (intentional, grave acts) and a broad definition (including forms of sloppiness). Because we found only very limited usage of terms referring to the culpability and intentionality of actions in the empirical research, we could not include the dimension of intentionality in our analysis, even though it was put forward by Fanelli as a key issue of contention of definitions (2011).

### Value-Based or Norm-Based

Besides the dimensions discussed by Fanelli, other grounds for the diversity in definitions of integrity and misconduct in research have been proposed. Godecharle et al. (2013) distinguish two main approaches to define integrity and misconduct. They use the terms ‘positive approach’ for those guidelines emphasizing the principles of research integrity, and ‘negative approach’ for those focussing on a definition of misconduct, i.e. breaches of integrity (Godecharle et al. 2013). In order to better understand the diversity in guidelines and codes of conducts, they translated the distinction between the two approaches into the ethical concepts of values and norms, distinguishing between a value-based and a norm-based approach to integrity, respectively (Godecharle et al. 2014). As they state, definitions of misconduct are based on norms: punishable misbehaviour implies a rule that is broken. They expect a larger diversity among norms and definitions of misconduct than positive notions of scientific values, as “the unavoidable differences in research contexts will lead to diverse definitions”. A value-based approach is more reliant on the underlying values of researchers, which are “more likely to be universally accepted” (Godecharle et al. 2014).

A similar distinction is made by Steneck (2006). Regarding several definitions of misconduct, integrity and responsible conduct of research, he distinguishes between those defining research integrity in terms of ‘moral principles’ and those defining it in terms of ‘professional standards’. The former is closely related to the value-based approach as indicated by Godecharle et al., whereas the latter resembles the norm-based approach. In his article, Steneck furthermore poses that “defining research integrity in terms of both moral principles *and* professional standards is problematic,” hence insisting on a choice of either of the two.

Not only does a distinction between the value-based and norm-based approach in defining misconduct and integrity lead to different definitions, it also has implications for the possible prevention of research misconduct and promotion of integrity, according to Godecharle et al. (2014). Adhering to a value-based approach might lead to a focus on training and the use of role models, whereas adhering to a norm-based approach would make one more likely to focus on generating clear and applied rules and potential sanctions. Even more generally, one might expect a report written in a norm-based (or ‘negative’) fashion to be more focused on the repression of misconduct, rather than on the promotion of ‘good science’ in the form of responsible conduct of research. The contrary might be expected from a document written in a value-based manner.

### Components of Research

There is no consensus about what components of the research process are considered to be relevant for integrity in science. In a 2005 study by Martinson et al., it was shown that the frequency of scientists engaging in questionable research practices widely exceeds the number of scientists engaging in FFP (Martinson et al. 2005). They therefore suggested that definitions of misconduct restricted to FFP are too limited, because only a small proportion of acts harming science are captured under this definition. For their analysis, they expanded the list of forms of misconduct beyond ‘data cooking’ to breaking rules about experimentation with human subjects, repeat-publication, inadequate record keeping, or failure to disclose conflicts of interest. Their list includes sixteen major forms of misconduct, covering a wide range of components of the research process. The list arose from focus-group discussions in which scientists from top-tier research universities commented on the misbehaviours that were of greatest concern to them (Martinson et al. 2005).

Considering the ‘ordinary language condition’ (Salwen 2015), it can be reasonably expected that these issues should be present to some degree in documents defining or discussing research integrity. To make the list more wieldy for our analysis, we grouped the sixteen issues into five broader categories covering major components of the research process (the numbers in brackets correspond to the numbering of the acts of misconduct in Martinson et al. 2005):

- Data management (1, 5, 6, 7, 9, 15)
- Human or societal aspects and personal contact (2, 4, 8)
- Authorship and publication (3, 5, 11, 12, 13)
- Funding of research (3, 10)
- Methodology of research (9, 10, 13, 14, 15, 16)

With this list, we do not aim to measure ‘broad versus narrow’, but to identify more specifically which kinds of components of the research process are being discussed or avoided.

Martinson’s list closely resembles the OECD’s (Organisation for Economic Cooperation and Development) categorisation of scientific misconduct (OECD 2010), apart from minor differences such as the absence of ‘personal misconduct’ (for which OECD refers to ‘inappropriate personal behaviour’ and ‘insensitivity to social or cultural norms’). In contrast, the OECD does not explicitly refer to ‘human or societal aspects’ in their categorisation, though ‘abuse of laboratory animals’ and ‘violation of human subject protocols’ are listed (under the heading ‘research practice misconduct’, which otherwise seems to refer to methodological aspects of research). Because of the systematic way in which Martinson’s list was created, we chose to base our own analysis on that one.

### Patterns in the Variety

As is clear, an overwhelming variety of definitions of scientific integrity and scientific misconduct is available and some of these exhibit major differences. Many efforts have been undertaken to form novel codes and guidelines for research integrity, sometimes with the ambition to definitively resolve this miscellany (Steneck 1999; Fanelli 2013).

In our analysis, we attempt to identify patterns in this variety, using the three dimensions we identified through the literature (broadness, value vs. norm-based, different components). Despite the debate on research integrity being presented as if there is only one shared debate we will show that there are major differences between the debates on research integrity within science, policy and media. They vary in broadness, each having its specific focus on various components of research, and preferred measures to resolve a lack of integrity.

## Methods and Data

The analysis makes use of co-word analysis, as developed in the context of scientometrics, the “quantitative study of science, communication in science, and science policy” (Hess 1997). Scientometrics is a methodological approach in which science itself becomes the subject of quantitative research. Originally, scientometrics started off as a tool to improve scientific information retrieval, using the Science Citation Index. However, it soon became a novel instrument in the empirical study of sciences and is currently used to measure scientific impact and citation scores, to map the growth and relations between research fields, or the position of universities in the research system (e.g. Leydesdorff and Milojevic 2015; Halffman and Leydesdorff 2010).

Co-word analysis is an established scientometric method. It maps how signifiers (i.e. words or phrases) occur together in texts, in order to identify relationships between concepts or shifting patterns in (scientific) language (Callon et al. 1991; Leydesdorff and Welbers 2011; Leydesdorff 2001). The strength of connections between signifiers is measured through various indices based on the frequency of co-occurrence. Using these indices, signifiers are clustered into groups with network maps. Such proximity maps can be used to reveal systematic connections between concepts that might not have been noticed by simply reading the documents, especially in large sets. These larger sets allow us to find subtle structures in the usage of the term ‘integrity’ and frequently co-occurring words and themes, as opposed to the detailed meaning of the terms that may be found by close, in-depth reading of texts. We recognise therefore, that this quantitative approach should not be regarded as a substitute for in-depth study of the text, but rather forms an extension of these methods for large sets of documents. The availability of adequate software tools and the ability to study the entire body of academic literature on scientific integrity constitute additional motivations to employ a quantitative approach.

By comparing network maps between different time periods, the dynamics of specific research subjects or concepts can be traced (Qin 1999; Leydesdorff and Milojevic 2015). In this way conclusions can be drawn on the relationship of different terms in the integrity debate and it can be shown what terms and concepts shape the debate in various periods and media.

Our analysis makes use of the *Jaccard index* as measure of proximity, using the quantitative content and text mining program *KH Coder* (Sourceforge.net 2016). For two signifiers X and Y the *Jaccard index* J(X, Y) is computed as:

$$J\left( {X,Y} \right) = \frac{\# (X\, and\, Y)}{\# (X\, or\, Y)} = \frac{\# (X \, and\, Y)}{\# \left( X \right) + \# \left( Y \right) - \# (X \, and\, Y)}$$

In this, $\# (X)$ is the number of units containing X, in which ‘unit’ refers to either a sentence or a paragraph (i.e. we count the number of times that signifiers X and Y occur in the same unit, relative to the number of units that contains either X or Y). Besides the Jaccard index, one of the major indices used in co-occurrence analysis (Tanimoto 1958; Qian et al. 2011), other indices were used, such as the cosine index and Ochiai coefficient. These other indices gave very similar results as those presented in this paper and were therefore omitted.

We searched for terms related to integrity and their (frequent) co-occurrence with other relevant terms, in order to analyse how research integrity was discussed. For this analysis, we selected English language documents from scientific journals, policy documents and newspaper articles, in order to compare discursive patterns in these three domains. In addition to an analysis of the contemporary usage, we also traced the genealogy of ‘integrity’ over time, using the documents’ publication dates. To some extent, our approach is similar to the one described by Walterbusch et al. (2014), in which they identified the genealogy and use of the word ‘trust’ in scientific literature over the past fifty years, or the work by Moretti and Pestre, tracing the development of the language used by the World Bank (Moretti and Pestre 2015).

### Scientific Texts

Scientific texts were collected from the *Web of Science* database, containing publications from all research fields by joining the Science Citation Index Expanded, the Social Sciences Citation Index, the Arts & Humanities Citation Index, and the Conference Proceedings Citation Index (Thomson Reuters 2016). Several sets of documents were selected:

1. *Abstracts of scientific publications* all publications that contain either of the phrases ‘scientific integrity’, ‘research integrity’, ‘scientific misconduct’, or ‘research misconduct’ in their title or abstract. This resulted in a set of 637 abstracts, used in the analysis. Furthermore, we specified three periods in order to study shifting patterns over time:
  1991–1995: 68 articles
  2001–2005: 86 articles
  2011–2015: 210 articles
2. *Top frequency articles* We performed a full-text analysis on *Web of Science* articles that contain the words ‘scientific integrity’, ‘research integrity’, and/or ‘scientific misconduct’ most frequently in their abstract. This set was used to validate the analysis of abstracts and titles only. The analysis made clear that, concerning their usage of terms related to integrity in science, the abstracts of scientific articles are representative of complete articles. Consequently, the amount of analysed abstracts provides validation and robustness to the results and conclusions presented in this article.
  There are ten articles in the database that contain at least five occurrences of either of the above three specified search terms. (These articles can be found in the *Web of Science* with an ‘advanced search’ using a ‘ut=’ query and the association numbers found in the supplementary material, part A.)
3. *Science and Nature publications* As a last set of data, we collected the publications in the *Web of Science* database that were published in *Science* or *Nature* and that contain the phrase ‘research integrity’ or ‘scientific integrity’ in their title, abstract or keywords. Articles from these journals were selected because *Science* and *Nature* are highly influential academic journals and have also been a prominent forum in the discussion of scientific integrity. The gathered articles were subdivided over three periods:
  OLD: period 1987–1990 (5 Nature, 7 Science, all that was available)
  MIDDLE: period 1995–2000 (10 Nature, 10 Science—selection based on most cited)
  NEW: period 2010–2015 (9 Nature, 8 Science, all that was available)
  For the first and last period, all articles matching the search terms were gathered, while from the abundance of the middle period only the most frequently cited articles were selected. This was based on the assumption that the articles with most citations have the highest probability of being influential on the discourse of integrity and with the intention to create sets of similar size. The periods were chosen to correspond to the set of policy documents, described below. The choice for the range of the first period was grounded on the availability of publications, starting in 1987.

### Policy Documents

Policy documents were collected from an inventory of 126 international key policy documents, established as part of the EU PRINTEGER project. The inventory includes the following information: organisation that produced the document, name of the document, date, region, target group, type of the document (e.g. guidelines, statement, report), whether following the policy stated in the document is mandatory, and whether the document describes procedures for dealing with alleged misconduct and is available online (PRINTEGER 2015).

From this list, two sets of documents were collected:

1. *Diachronic analysis* To analyse change over time, 20 documents were collected: the most recent ten documents and the oldest ten documents from the inventory. The most recent ten documents all date from 2014 or 2015 (6 from 2014, 4 from 2015), while the oldest ten documents date from the period 1995–2001 (1 from 1995, 1 from 1997, 1 from 1998, 2 from 1999, 3 from 2000, 2 from 2001).
  From the recent documents, six were classified as ‘(university) policy’, three were classified as ‘guideline’ and one as ‘recommendation’. The oldest documents were classified as ‘guideline’ (4), ‘(university) policy’ (4), ‘declaration’ (1), and as ‘rules for conduct’ (1).
2. *International comparison* Policy documents from five European countries were collected: Germany, Italy, Norway, the Netherlands, and the UK. A specific focus on European documents was chosen because of the European nature of the PRINTEGER project, for which part of the research was performed. In addition, qualitative analysis of policy documents in the PRINTEGER framework demonstrated that policy statements might differ, among others, between northern and southern European countries, due to their culturally different background (PRINTEGER 2016). To respect these differences, to gather a representative sample and to build upon the previous work in PRINTEGER, we chose to use policy statements from the countries specified above.
  In order to gather a uniform set of data, we selected the university and institute policies (hence only policy documents specific for one research institute or university and not those from national policies or umbrella organisations) that were available in English. Documents were gathered from the PRINTEGER inventory (PRINTEGER 2016). This resulted in a set of 10 British documents, 9 Dutch documents, 8 German documents, 4 Italian and 5 Norwegian documents. To avoid effects from changes over time, all documents in this set were gathered from the period after 2002, with the vast majority of documents dating from after 2010.

#### Newspaper Articles

Newspaper articles were gathered through the LexisNexis search engine for English language newspapers (PRINTEGER 2016). Articles were retrieved via the search term “‘research integrity’ OR ‘scientific integrity’” for the title or lead of the article. We then divided the search results in three periods, matching those of the periods used in the analyses of the scientific publications:

OLD: 15 articles from 1987 to 1990
MIDDLE: 19 articles from 1995 to 2000
NEW: 19 articles from 2010 to 2015

In the first phase, the OLD phase (1987–1990), fifteen articles matched our search terms and we selected them all (after checking they were actually about research integrity). In the MIDDLE and NEW phases, there were more articles that matched the search terms. We sorted these articles by relevance (a standard option in *LexisNexis*) and deleted duplicates and articles that seemed too far off-topic, resulting in 19 articles for both periods.

#### The Analysis

The data were prepared for analysis by a spelling check, as misspelled words or differences in British and US spelling lead to imprecise and potentially invalid results. Similarly, hyphenation was removed, as the software accepts compound words with dashes, but it cannot differentiate between dashes and hyphens. Pre-processing also required that all plurals were brought into singular and all verbs were transformed to the present tense. In addition, so-called *stop words* were removed. These are trivial words or phrases that are commonly used in texts and that are of minor importance for content analysis, such as ‘the’, ‘and’, ‘or’, ‘as’ etc. The standard list of stop words of *KH Coder* was extended with the words ‘c’, ‘p’ and ‘%’. Finally, all terms were given a tag identifying them as ‘nouns’, ‘verbs’, ‘adjectives’, ‘adverbs’, or ‘proper nouns’.

After preparing the data for the analysis, we analysed co-occurrence networks between *signifiers*, followed by an analysis of co-occurrence between *themes* by grouping signifiers into broader categories. Co-occurrence networks of specific signifiers were made for co-occurrence on the level of sentences and of paragraphs. Unless otherwise stated, all co-occurrence networks represent the sixty strongest connections, measured by the Jaccard index. In these figures, a line (in this field of research usually called ‘edge’) between two words indicates that these two words are among the sixty strongest connections. The choice to use sixty connections is a default setting of the software used (Sourceforge.net 2016) and results from balancing the inclusion of significant words and the clarity of the resulting diagram. Thicker edges correspond to stronger connections. A cluster of connected words is given the same colour and larger nodes correspond to more frequently used words. The positioning of the words does *not* indicate a level of connection between them. Nodes that are nearby, but not connected by an edge do, in general, not represent a stronger connection between the occurrences of the corresponding words, than nodes that are further apart. Because of the high dependence on the origin of the studied documents, ‘proper nouns’ are not included in the networks except for the proper noun ‘Integrity’. Hence the term ‘integrity’ may occur twice in a network, once as a noun referring to the concept of integrity and once as part of a name of an institute, office etc. These cases can be distinguished by the fact that the latter will be written with a capital, whereas the former will not. Occurrences of the term ‘integrity’ as the first word of a sentence (and for this reason written with a capital) are counted as a noun, rather than a proper noun.

For the analysis of themes, we grouped signifiers into categories that refer to common phenomena, listed in Table 1. In this, the data analysis goes beyond a plain quantitative co-word analysis, but includes a qualitative layer: different keywords are coded, based on their meaning, rather than just counting the signifier. We grouped synonyms and words with similar meaning and connotations into categories of *themes*. Thereby we aimed to unify the analysis and compensate for the potential plurality of terms referring to the same concept. For example, the words ‘article’, ‘publication’ and ‘paper’ all refer to the same concept and are therefore grouped under the same theme. A complete list of the themes and all words that have been categorised under them can be found in the supplementary material. The classification of the themes was done primarily inductively, by considering the word lists and frequency tables of a set of ten policy documents and ten scientific publications, using techniques described by Ryan and Bernard (2000). Subsequently, words were selected by hand and classified under the identified themes. Again using the Jaccard index and the same representational conventions, we then produced co-occurrence networks for themes, representing the twenty strongest connections.

**Table 1:** Classification of words into themes

Theme	Examples of words
Authorship	Author, authorship, journal, paper, publication, publish
Education	Education, training, train, educate
Finance	Fund, funding, grant, finance, tax, money, cost
Institution	Institution, federal, community, national, government
Integrity	Integrity, ethical, ethic
Misconduct	Misconduct, plagiarism, fraud, fabrication, falsification
Policy	Policy, guideline, code, recommendation
Promote	Promote, protect, development, improve, ‘best practice’
Repression	Sanction, punish, corrective, accuse, allegation
Science	Science, scientist, research, researcher, academic
Society	Society, public, environment, health, human
Virtue	Trust, honesty, trustworthy, responsibility, respect, faith, dignity

## Results: Discursive Patterns of Integrity

In this section the results of our analyses will be presented. This will include references to various figures presented in the text and some figures available in the supplementary material. The discussion in this section will first concentrate on the contemporary differences between the integrity language in various media. Second, the evolution of these differences over time will be discussed. Lastly, this section provides an overview of the debate on integrity in policy statements from various countries. A summary of the results presented in this section, as well as remarks on what to conclude from this, will be provided in “Conclusion: Change and Discrepancy in Integrity” section.

### Contemporary Differences

**Fig. 1:**
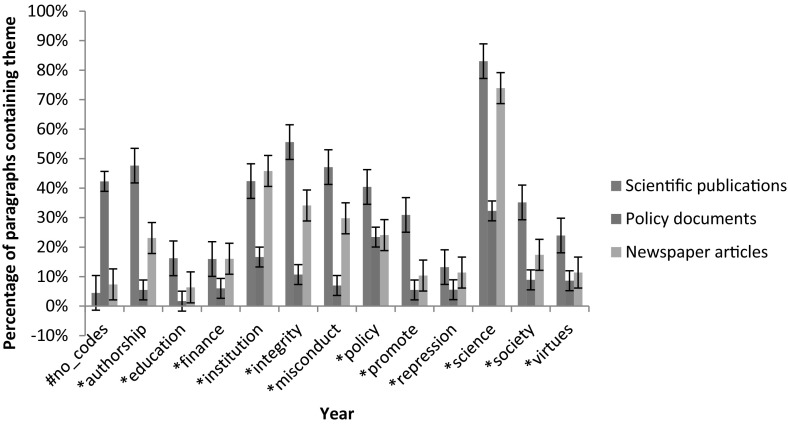
Theme analysis on articles from 2011 to 2015 period, with standard error margins

Figure 1 presents the results of the theme analysis on the most recent documents in all media. It shows clear differences between the various media. Besides several obvious differences, the figure clearly shows more attention for the promotion of good science in scientific publications as well as a prominent focus on authorship in the integrity debate in this medium.

**Fig. 2:**
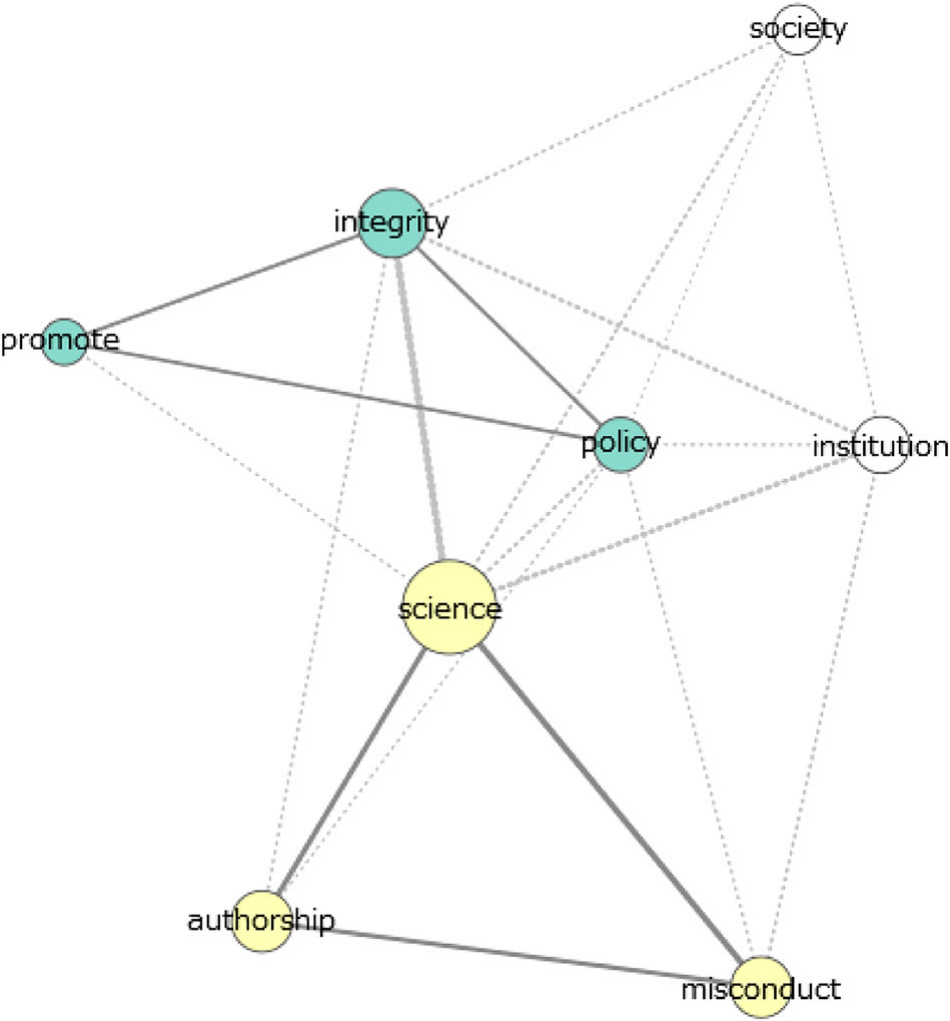
Co-occurrence network of themes in abstracts of 2011–2015 scientific publications

**Fig. 3:**
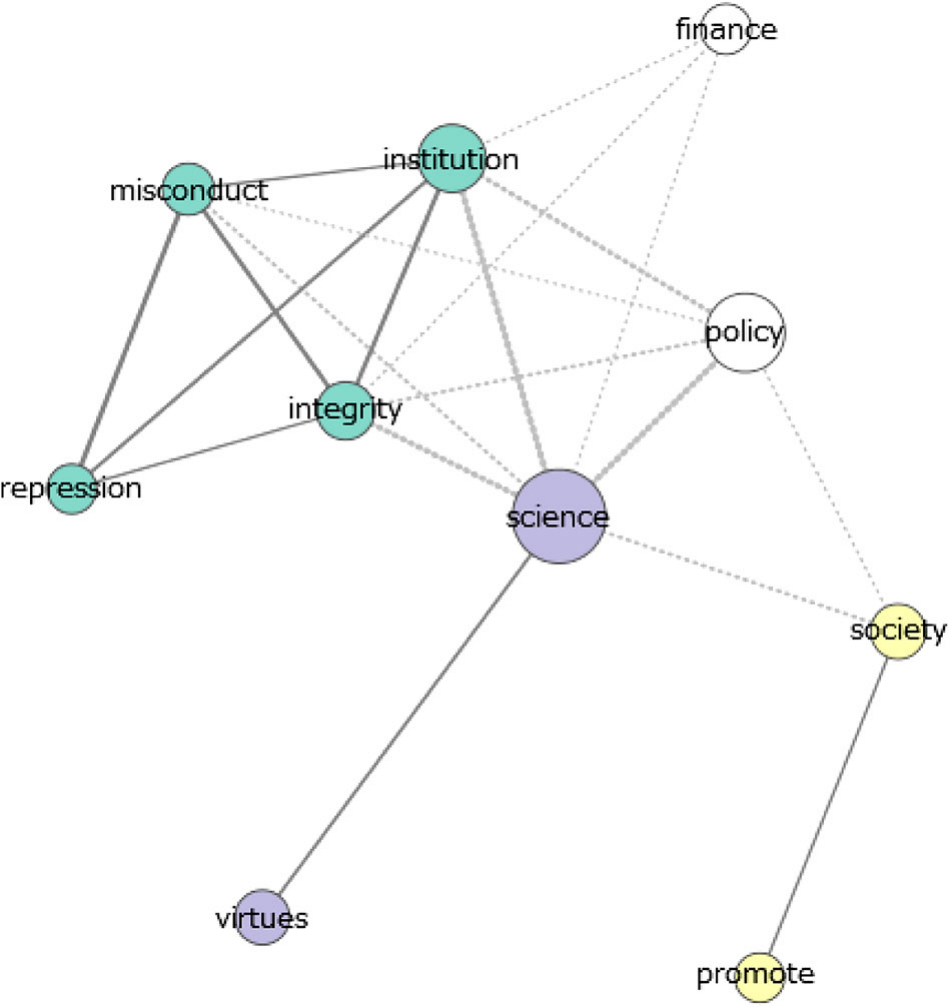
Co-occurrence network of themes in full-texts of 2011–2015 policy documents

**Fig. 4:**
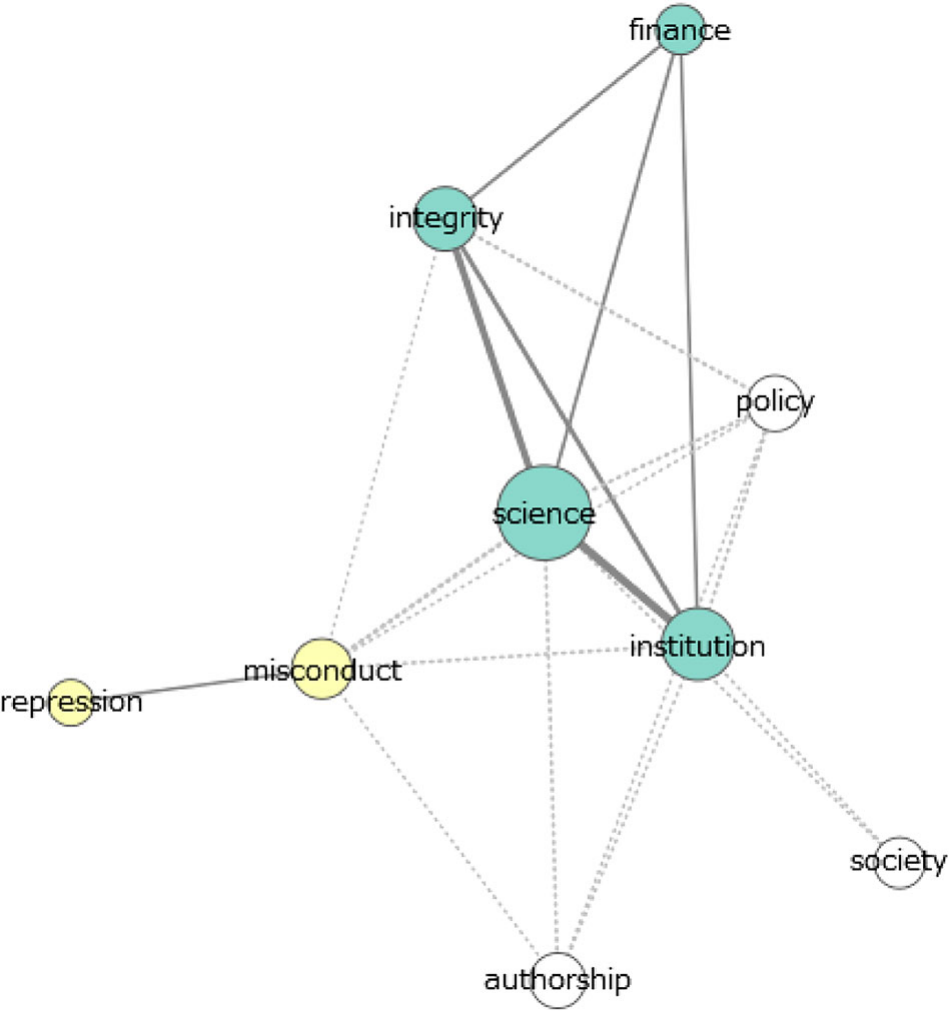
Co-occurrence network of themes in 2011–2015 newspaper articles

In addition, Figs. 2, 3 and 4 show the relationship, in terms of co-occurrence, between the various themes. They show the various themes and the twenty strongest connections between them in the most recent documents of scientific publications (Fig. 2), policy documents (Fig. 3) and newspaper articles (Fig. 4).

The data from these figures will be discussed on the basis of the dimensions indicated in the section “Theoretical Framework: The Dimensions of Integrity”. In this we stress that the above figures only present results of the theme analysis. The results of the analysis of individual signifiers can be found in the supplementary material. In several cases we will refer to these analyses, providing more detailed extensions of the results shown in Figs. 1, 2, 3 and 4.

#### Promotion Versus Repression

Regarding the adopted approach (value- or norm-based) in the texts, clear distinctions can be spotted between the scientific publications and policy documents. Whereas the former are characterised by frequent usage of terms referring to values, virtues and the focus on promoting integrity (as becomes clear from Fig. 1), the latter show a more frequent usage of terms referring to describing and punishing misbehaviour in research (as we conclude from Fig. 1 and from the strong connection between ‘integrity’ and ‘repression’ and ‘misconduct’ in Fig. 3). In scientific publications, the theme ‘integrity’ is closely related to the theme ‘promote’. In the policy documents, this connection shifts to the pair ‘repression’ and ‘misconduct’. Besides having a closer connection to integrity, the themes ‘promote’ and ‘virtues’ are, more frequently used in scientific publications compared to policy documents. For the pair ‘misconduct’ and ‘repression’ this difference is smaller, thus indicating a relatively higher usage of these themes in policy documents.

#### Components of Research

**Fig. 5:**
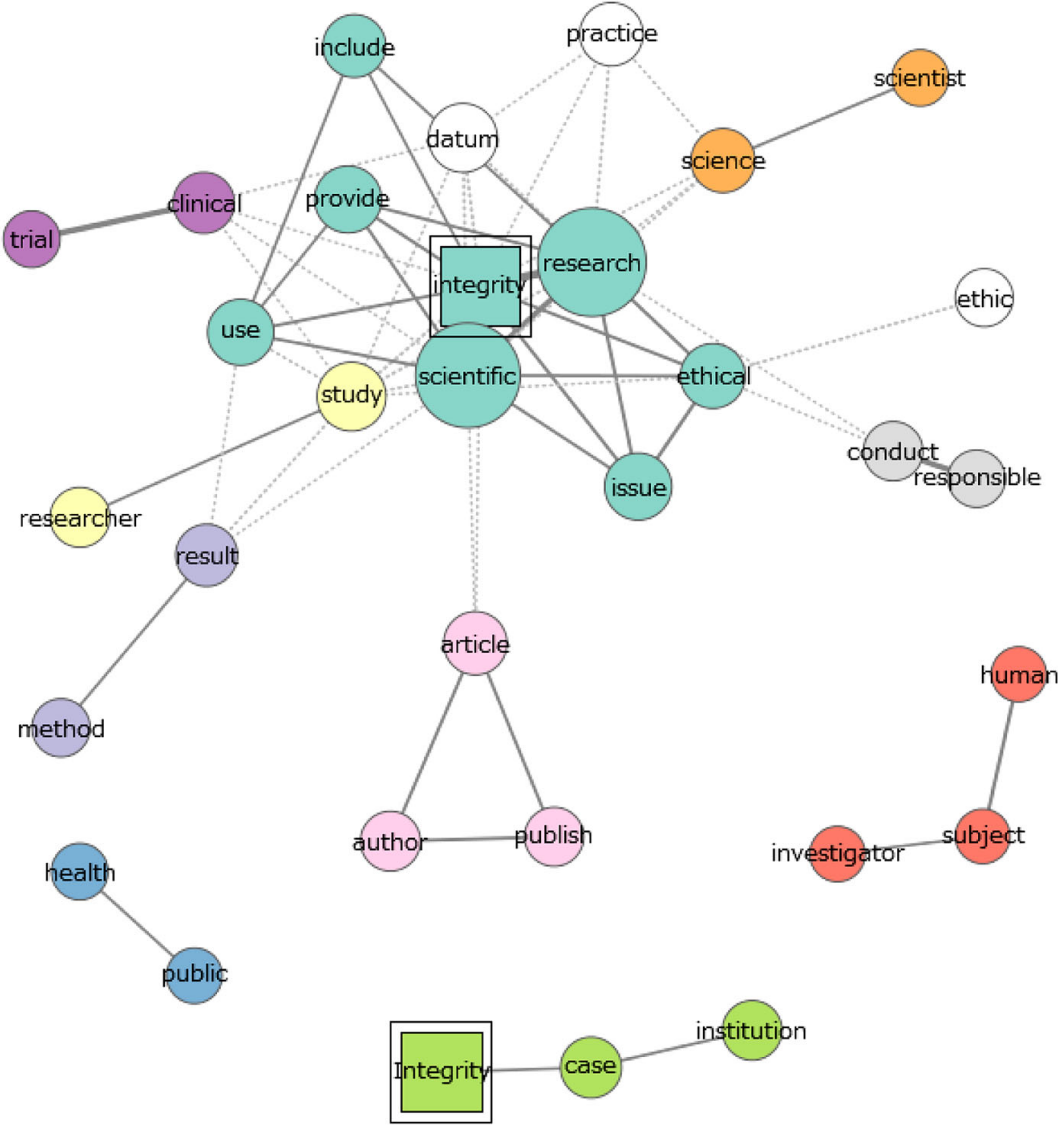
Co-occurrence network of ‘integrity’ in the abstracts of all scientific publications

Major differences can also be spotted in the various components of research that are linked to the concept ‘integrity’. Scientific articles tend to refer to the concept of authorship and publication most often (Fig. 1), while having substantial attention for methodology and (to a lesser extent) society. This can be concluded from the theme analysis, frequency analysis of individual words and co-occurrence networks of the term ‘integrity’, the latter of which are available in the supplementary material. Indeed, authorship is a frequently occurring theme in scientific documents and individual terms like ‘author’, ‘publish’ and ‘article’ have a close connection to the term ‘integrity’ in the co-occurrence network of the individual term ‘integrity’ (figure C14). Moreover, the frequent occurrence of words like ‘method’, ‘result’ and ‘clinical’-‘trail’ (in Fig. 5) show the attention for methodology in science. Lastly, the theme ‘society’ is used fairly frequently and clusters of individual terms like ‘important’-‘role’-‘society’, ‘health’-‘public’ and ‘public’-‘attention’ in the various figures demonstrate clear attention for societal aspects of science.

In both the policy documents and newspaper articles, a clear focus on financial and funding aspects of research is identified. Indeed, the theme ‘finance’ gains in attention over the years and the connection between the theme ‘integrity’ and the theme ‘finance’ undoubtedly gains in strength (most clearly in newspaper articles, concluding from Figs. 3 and 4 as well as figures C8–C10 in the supplementary material). These patterns are confirmed by the analysis of individual terms, with the presence or absence of terms like ‘money’, ‘fund’ and ‘taxpayer’ (in figures C1 and C2). In addition, a clear reference to authorship is missing, most notably in policy documents (for example concluding from Fig. 1).

With regard to the component ‘data management’, we note that the term ‘data’ (for instance in ‘data’, ‘database’, ‘data analysis’, ‘data storage’, etc.) is hardly used in all the documents. Hence, there is only limited explicit reference to this component of research in all media, despite it being put forward as a major aspect of concern by some commentators (Fanelli 2011).

#### Narrow Versus Broad

For the analysis along the dimension of broadness, we studied the co-occurrence networks of individual words. The co-occurrence network for the scientific documents is presented in Fig. 5. The co-occurrence networks for the other documents can be found in the supplementary material, part C (figures C1 and C2).

The figure shows the terms ‘ethical’ and ‘ethic’. In particular, the terms ‘integrity’ and ‘ethical’ have a strong direct connection. This indicates that the concept of ‘integrity’ is frequently used together with the concept ‘ethics’, suggesting that ‘integrity’ is discussed in a broad context including general ethics of science.

On the contrary, the term ‘integrity’ is closely connected to terms directly referring to misconduct such as ‘misconduct’ and ‘breach’ in recent policy documents (in the co-occurrence network of ‘integrity’ of policy documents, C1). In addition, words referring to specific forms of misconduct like ‘plagiarism’ and ‘fraud’ show up in the co-occurrence networks of ‘integrity’ of all recent policy documents, both in the temporal as well as the geographical distribution. This suggests a usage of the term ‘integrity’ as the opposite of misconduct and a usage in the more narrow sense of the term. This is especially the case in the recent documents. The older documents show less signs of narrow definitions, lacking the specific connections between ‘integrity’ and ‘misconduct’ and showing less frequent usage of the terms referring to specific types of misconduct.

For the newspaper articles, no clear indication can be found from our analysis for either very narrow or for more broad definitions.

### Evolution of Differences

The previous results show clear distinctions between the usage and understanding of the term ‘integrity’ in contemporary documents from various media. However, these distinctions have not always been so clear. This section presents the evolution of the usage of the term ‘integrity’. For the sake of brevity, the figures of the other temporal periods are omitted here. *They are presented in the supplementary material, part C (figures C3 to C14).*

#### Promotion Versus Repression

The theme analysis shows differences between recent policy documents and scientific publications. However, the differences between the older policy documents and the scientific articles are far less pronounced. In general, we note that the usages of the various themes in scientific articles are very constant throughout the various periods. For most themes, only very limited differences can be spotted between the frequencies in the older documents and the most recent ones (concluding from Fig. 1 and figures C3). The policy documents show more differences, especially with respect to the dimension promotion versus repression (figure C4). The older policy documents clearly show more frequent usage of the themes ‘virtues’ and ‘promotion’ than the recent policy documents. In addition, the connection between these themes and ‘integrity’ is much stronger (Fig. 3 and figure C9). Therefore, the older policy documents closely resemble the scientific documents with respect to the promotion–repression dimension.

This also becomes evident from the co-occurrence networks of individual terms. We find words such as ‘trust’, ‘dignity’, ‘respect’, ‘responsible’, ‘responsibility’ and ‘caring’ in older policy documents and scientific articles (figures C11–C14), while more recent policy documents refer to sanctioning and punishing misbehaviour in research (with terms like ‘failure’, ‘concealment’, ‘unreasonable’, ‘correction’ and ‘sanction’), as is shown in figure C1.

In newspaper articles, the adopted approach does not show as clearly as in the other documents. Nevertheless, a more value-based approach can be spotted in the older documents while in the more recent newspapers a slightly more norm-based approach can be identified in the theme analysis of these documents.

#### Components of Research

Last, we identified the most frequently mentioned components of research in the given documents, following the components identified in the theoretical framework. Again, the scientific articles show little change in the components discussed between different periods. However, in both the policy documents and newspaper articles, a shift of attention from societal aspects to financial and funding aspects of research occurs. Indeed, the theme ‘society’ received substantial attention in the older documents (both in newspapers as well as in policy documents, see figures C4 and C5), but this attention declines when passing to the more recent documents. In contrast, the theme ‘finance’ gains attention over the years and the connection between the themes ‘integrity’ and ‘finance’ clearly gains in strength (most clearly in newspaper articles, see figures C9 C10 and Fig. 4). These patterns are confirmed by the analysis of individual terms, with the presence or absence of terms like ‘patient’-‘health’, ‘money’, ‘fund’ and ‘taxpayer’ in figures C1, C2 and C11.

Strikingly, issues of authorship receive substantial attention in the scientific literature, but receive far less attention in the policy documents. This is the case for all periods as becomes clear from Fig. 1 and figures C3–C5)

### Geographical Comparison

**Fig. 6:**
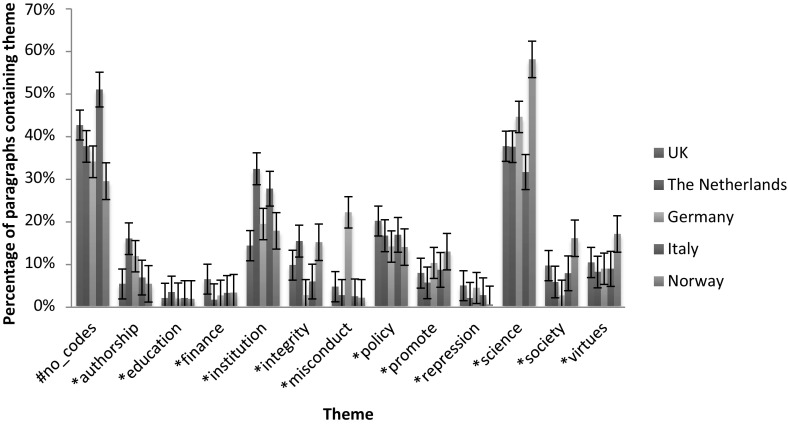
Theme analyses in policy documents, geographical distribution, including standard error margins

Next, the results of analyses on policy documents originating from different European countries will be presented. The documents were sampled from institutions in: Germany, Italy, Norway, The Netherlands and the UK. Figure 6 shows the usage of the various themes in policy documents from these countries.

First, we note some differences between the usage of the theme ‘integrity’ compared to the theme ‘misconduct’. Mainly in Dutch and Norwegian texts, the former theme is used in considerably more paragraphs than the latter. In German documents however, we spot a nearly tenfold higher usage of the theme ‘misconduct’.

A second striking feature is the usage of the theme ‘society’, which is remarkably low in German texts and remarkably high in Norwegian documents.

Furthermore, a relatively frequent usage of the theme ‘institution’ can be spotted in Dutch and Italian documents. Lastly, the extremely low attention for ‘repression’ in Norwegian texts is striking. These factors all point to differences within the discussion and hence, perhaps, culture on integrity in the different countries. This may not be considered surprising (given for example Godecharle et al. 2013; PRINTEGER 2016), though it is potentially worrying given the close collaboration among European scientists.

These trends are also visible in, and hence confirmed by, the co-occurrence networks of themes for the various countries. For reasons of clarity and brevity, these figures are omitted here, and can be found in the supplementary material, part C (figures C15–C19). Focusing on the role of the theme ‘integrity’ in the figures, we conclude that ‘integrity’ has a strong connection to ‘policy’ and ‘institution’ in the British, Dutch and Norwegian documents. However, it is has strong connection to ‘society’ in German policy documents and is connected to ‘virtues’ and ‘institution’ in Italian documents. In addition, there is a strong connection between ‘authorship’ and ‘integrity’ in the Dutch documents. Moreover, the themes ‘virtues’ and ‘integrity’ are connected in the network of Norwegian documents.

Furthermore, the theme ‘misconduct’ co-occurs most frequently with the theme ‘repression’ in British documents, with ‘institution’ and ‘science’ in the German documents and—remarkably—with ‘virtues’ in Dutch texts. In the other figures, the theme ‘misconduct’ is not present (showing that it is only weakly connected to other themes in the Italian and Norwegian documents).

Last, we analysed the usage of individual words within the selected documents. The co-occurrence networks are presented in the supplementary material, Part C (figures C20–C24). The term ‘integrity’ shows up only in the co-occurrence networks for the Netherlands, the UK and Norway, indicating that the term has no particularly strong connections to any other term in the German and Italian documents. In the Norwegian and British documents we observe that it is connected to ‘research’ and ‘researcher’. Most interestingly, the term ‘integrity’ is strongly connected to ‘violation’ and ‘complain’ in the Dutch document, showing clear attention for a lack of integrity in research and a negative approach towards the phenomenon.

Furthermore, we note that the term ‘honesty’ shows up in three of the five figures (missing only in the Dutch and German figures). In addition, we spot the words ‘fairness’, ‘impartiality’ and ‘equity’ in the Italian figure, showing attention for virtues in these documents that we discovered earlier in the theme analysis.

As might have been expected regarding the previous analysis, there is fairly little reference to the concept ‘authorship’ in the policy documents of the various countries.

Summarising, we spot clear differences between approaches to integrity in the policy documents of the various countries. Most prominently, an adherence to a value-based, positive approach in Norwegian and Italian documents can be identified, while mainly German and Dutch documents tend to demonstrate a norm-based, negative approach (see also Godecharle et al. 2014; Steneck 2006). In addition, different focuses on the various components of research can be identified, with for example the focus on authorship in Dutch documents and societal aspects in Norwegian documents. These findings confirm the conclusions on the heterogeneity of European policy documents on scientific integrity and misconduct from Godecharle et al. (2013, 2014).

## Conclusion: Change and Discrepancy in Integrity

After the term ‘integrity’ gained currency in the scientific arena in the 1980s, the meaning of the term, as well as its usage, have been subject to heated debate. The dimensions of this debate can be identified on the basis of reflections by prominent researchers and commentators on this issue. In these reflections, integrity is generally presented as a joint concern for scientists, research organisations and policymakers alike. However, including the full range of written documents on this topic, we have observed that this debate is by no means a singular discourse, but rather that it has followed different lines in different media. In scientific publications, integrity is considered a value and a virtue, closely related to ethics in research and hence placing integrity in a broad context of science ethics. As such, integrity is presented as the basis of ‘good scientific practices’ that should be promoted. Moreover, ‘integrity’ is frequently associated with the debate about authorship and what it means to be an author in the scientific publications, compared to the other media.

In science policy documents, the term ‘integrity’ has gradually lost its connection with ethics and is currently used in a more narrow fashion. In contrast to scientific publications, the term ‘integrity’ has become more directly used as the opposite of ‘misconduct’ in science policy documents. The concept of ‘integrity’ is increasingly approached from a repressive and norm-based perspective in policy documents, stressing the need to prevent misconduct through clear rules and providing ways of sanctioning behaviour that breaks these rules.

The patterns in newspapers are less pronounced than in the other media. There are no clear indications for the employment of either extremely narrow or broad definitions. However, we can spot a gradual shift from a value-based approach towards a norm-based approach, and from a focus on societal aspects of science towards a focus on financial aspects. As such, newspaper articles tend to hold the middle between scientific publications and policy documents, even though the contemporary discussion in newspapers resembles the discourse in policy documents more closely than that in scientific articles.

The approaches between the two major media in science, scientific publications and policy documents, seem to be diverging, showing major shifts in attention and approach in only two decades. The results of our research are summarised in Table 2. Because of the major discrepancies between the older and more recent policy documents we separated the policy documents in two classes.

**Table 2:** Dimensions of differences between the concept of ‘integrity’ in various media

Documents	Narrow versus broad	Value- versus norm-based	Aspects of research
Scientific publications	Broad	Value-based	Authorship
			Methodology
			Society
Older policy documents	Broad	Value-based	Society
			Methodology
Recent policy documents	Narrow	Norm-based	Finance
Newspaper articles	No clear indication for either	Minor shift from value- to norm-based	Shift from society towards finance

Undoubtedly there are several connections between the various media studied. As became clear from the diachronic analysis, coverage of the concept of research integrity in newspapers is nourished by input from scientific publications. In addition, as with many policy aspects, the formation of novel policy documents, including codes of conducts and guidelines, is likely to be dependent on the reporting of (severe) cases of misconduct and the outcry that it creates. Among others, newspaper articles can be considered as one of the major forms of such reporting. As a last part of the cycle, several scientific articles have been published that specifically comment on novel policy documents, their strengths and their weaknesses (e.g. Salwen 2015). Hence, the various media nourish each other, with mutual comments and references. Considering these interrelations, it should be deemed even more surprising that the approach to the concept of integrity varies so widely between the distinct media.

Even between countries, there are noticeable differences in how integrity is addressed in the policy arena. Not only does policy respond to incidents that are particularly salient in a national context with diverging timing of policy initiatives, but also with concerns and approaches that seem to follow national interests and policy styles as well as more universal concerns about research.

Concluding, we identified differences in the approaches towards integrity in multiple dimensions: differences occur on a temporal basis with diverse approaches in distinct phases; they occur on a geographical basis with discussions in distinct countries following their own national trends and specific focus points; and differences occur between the various media, each having its own specific discourse.

Harmonising approaches to integrity or imposing one definition of integrity may well be too disrespectful of the diversity in national styles, research practices, or indeed of normative and political disagreement about what constitutes the gravest issues of concern for research. However, there is one particular disparity we find worrying. Based on our results, it appears that especially the discourses of scientists and policymakers are diverging increasingly. Whereas scientists discuss integrity as a broadly defined virtue that should be promoted, with specific attention to authorship issues, policy documents seem to be following a more regulatory tone, with rules based on narrow definitions and with greater attention for financial concerns. In our opinion, these discrepancies are problematic and present several dangers to improved integrity: scientists may come to see integrity policy initiatives as increasingly alien, not addressing their key concerns (such as authorship). Policies that do not connect with the concerns of those involved face implementation problems, such as obstruction and ritual compliance. In addition, it is doubtful whether rules alone will actually improve integrity, as external rules do not become native easily to professional cultures and in this case seem to focus on only a limited range of actual integrity issues. Ultimately, these discrepancies may contribute to a polarisation between researchers and those that seek to guide or manage them. In the end, major discrepancies between the focus of those setting and formulating the norms and those having to live up to them can hardly be considered advisable. Therefore, we recommend that science policymakers should adjust their approach towards defining and discussing the concept of integrity to connect more closely with the daily focus and practices of scientists. We believe that this might be a first, and rather achievable, way to foster integrity in science.

## Electronic supplementary material

Below is the link to the electronic supplementary material.

Supplementary material 1 (DOCX 1478 kb)
