# Supplementary material for: Promoting Virtue or Punishing Fraud: Mapping Contrasts in the Language of ‘Scientific Integrity’
Source: Sci Eng Ethics. 2016 Dec 19;23(6):1461–85. doi: 10.1007/s11948-016-9858-y (PMC5705733; doi:10.1007/s11948-016-9858-y)
Supplement: Supplementary file 1 — Supplementary material 1 (DOCX 1478 kb) [file 11948_2016_9858_MOESM1_ESM.docx]

# Supplementary material

**Title**Promoting virtue or punishing fraud: mapping contrasts in the language of ‘scientific integrity’

**Journal**Science and Engineering Ethics

**A: Association numbers**

The ten articles in the database that contain at least five occurrences of either of the three specified terms (‘scientific integrity’, ‘research integrity’, and/or ‘scientific misconduct’) can be found in the *Web of Science* with an ‘advanced search’ using a ‘ut =’ query and the following association numbers:

| Article  1 | Association number  000327911100017 |
| --- | --- |
| 2 | 000355007600012 |
| 3 | 000167244200003 |
| 4 | 000331980900005 |
| 5 | 000339333600008 |
| 6 | 000301580600004 |
| 7 | 000326893700006 |
| 8 | 000334366300001 |
| 9 | 000286804500001 |
| 10 | 000227474900009 |

**B: Classification of theme analysis**

The following coding was used for the theme analyses. Words in bold, proceeded by *, indicate the name of the theme. The phrases below, separated by |, indicate the words classified under the theme.

***integrity**integrity | ethic | ethical

***misconduct**misconduct | fraud | plagiarism | plagiarize | fabrication | falsification | fraudster | fraudulent | fraudulently | fabricate | falsify

***science**science | scientific | scientist | research | researcher | academic | university

***finance**fund | funding | funder | funded | grant | tax | taxpayer | money | cost | costs | costly | financially | finance | financial | business | budget

***education**educate | training | train | education

***society**society | societal | human | environment | animal | environmental | environmentally | public | publicly | health | patient

***authorship**author | coauthor | co-author | authorship | journal | publication | publish | published | article | publisher | publishing | publish-or-perish | manuscript | editor

***virtues**trust | trustworthy | trusting | responsible | responsibility | dignity | respect | faith | confidence | honest | honesty

***institution**institution | institutional | community | university | committee | agency | national | european | federal | federally | government | department

***repression**sanction | corrective | retract | retraction | allegation | punish | punishment | accuse

***promote**promote | promotion | promoting | protect | protection | develop | development | improve | good | (highest & standard) | (best & practice) | strengthen

***policy**policy | guideline | recommendation | report | procedure | statement | code

**C: Graphs and figures**

Below we present the figures and tables that are referred to in chapter four. Figures are ordered by the occurrence of their references in the text.

List of figures:

| Figure number | Figure description |
| --- | --- |
| C1 | *Co-occurrence network of ‘integrity’ in NEW(2014-2015) policy documents* |
| C2 | *Co-occurrence network of ‘integrity’ in NEW (2010-2015) newspaper articles* |
| C3 | *Theme analysis of abstracts of scientific articles, including standard error margins* |
| C4 | *Theme analysis in policy documents, temporal division, including standard error margins, for the periods OLD (1995-2001) and NEW (2014-2015)* |
| C5 | *Theme analysis in newspaper articles, including standard error margins for the periods OLD (1987-1990), MIDDLE (1995-2000) and NEW (2010-2015)* |
| C6 | *Co-occurrence network of themes in abstracts of 1991-1995 scientific publications* |
| C7 | *Co-occurrence network of themes in abstracts of 2001-2005 scientific publications* |
| C8 | Co-occurrence network of themes in full-texts of OLD (1995-2001) policy documents |
| C9 | *Co-occurrence network of themes in OLD (1987-1990) newspaper articles* |
| C10 | *Co-occurrence network of themes in MIDDLE (1995-2000) newspaper articles* |
| C11 | *Co-occurrence network of ‘integrity’ in OLD (1995-2001) policy documents* |
| C12 | *Co-occurrence network of ‘integrity’ in the abstracts of 1991-1995 scientific publications* |
| C13 | *Co-occurrence network of ‘integrity’ in the abstracts of 2001-2005 scientific publications* |
| C14 | *Co-occurrence network of ‘integrity’ in the abstracts of 2011-2015 scientific publications* |
| C15 | *Co-occurrence network of themes in British policy documents* |
| C16 | Co-occurrence network of themes in Dutch policy documents |
| C17 | *Co-occurrence network of themes in German policy documents* |
| C18 | *Co-occurrence network of themes in Italian policy documents* |
| C19 | *Co-occurrence network of themes in Norwegian policy documents* |
| C20 | *Co-occurrence network of ‘integrity’ in British policy documents* |
| C21 | *Co-occurrence network of ‘integrity’ in Dutch policy documents* |
| C22 | *Co-occurrence network of ‘integrity’ in German policy documents* |
| C23 | *Co-occurrence network of ‘integrity’ in Italian policy documents* |
| C24 | *Co-occurrence network of ‘integrity’ in Norwegian policy documents* |

**Contemporary differences**


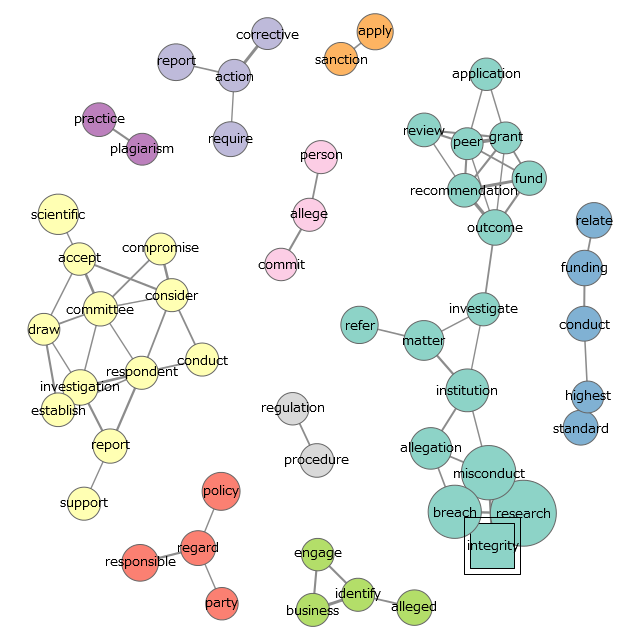


Figure C1 *Co-occurrence network of ‘integrity’ in NEW(2014-2015) policy documents*


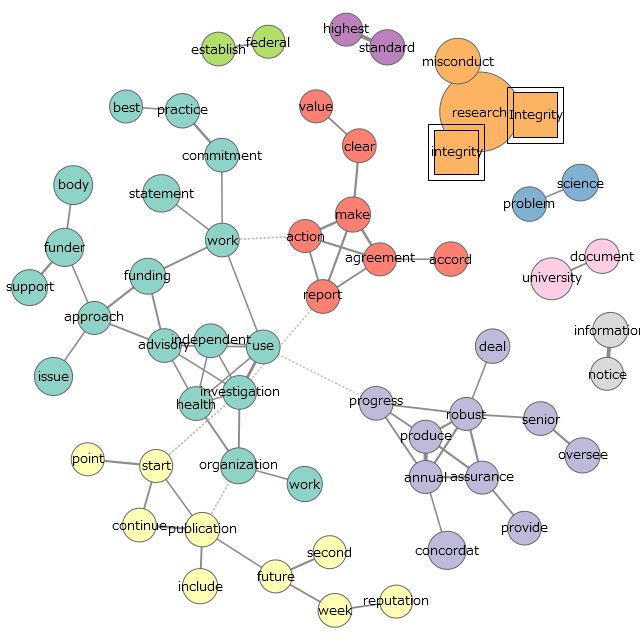


Figure C2 *Co-occurrence network of ‘integrity’ in NEW (2010-2015) newspaper articles*

Evolution of differences

Figure C3 *Theme analysis of abstracts of scientific articles, including standard error margins*

Figure C4 *Theme analysis in policy documents, temporal division, including standard error margins, for the periods OLD (1995-2001) and NEW (2014-2015)*

Figure C5 *Theme analysis in newspaper articles, including standard error margins for the periods OLD (1987-1990), MIDDLE (1995-2000) and NEW (2010-2015)*


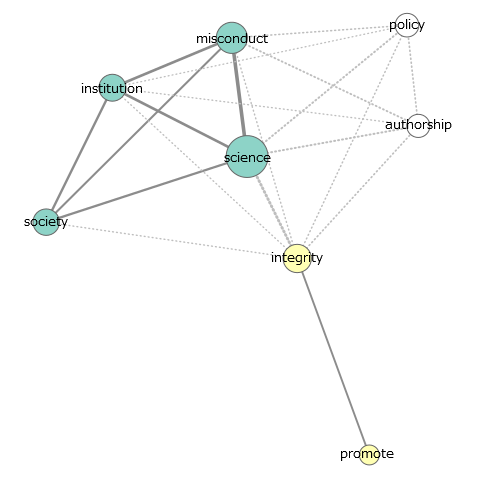


**Figure C6 *Co-occurrence network of themes in abstracts of 1991-1995 scientific publications***


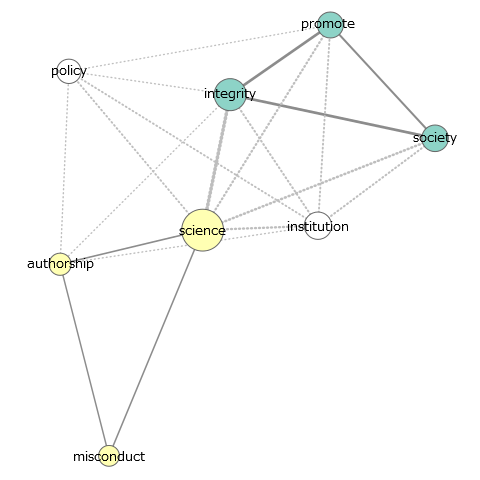

Figure C7 Co-occurrence network of themes in abstracts of 2001-2005 scientific publications


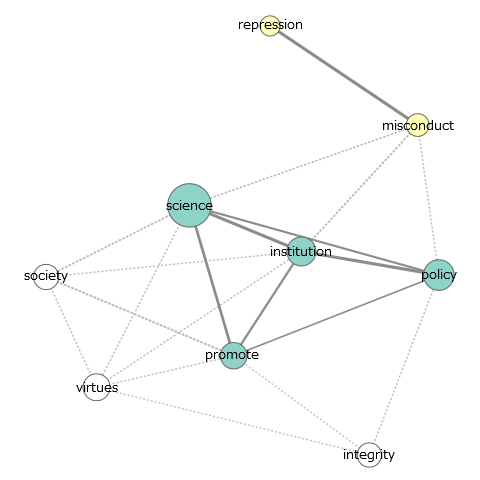


Figure C8 Co-occurrence network of themes in full-texts of OLD (1995-2001) policy documents


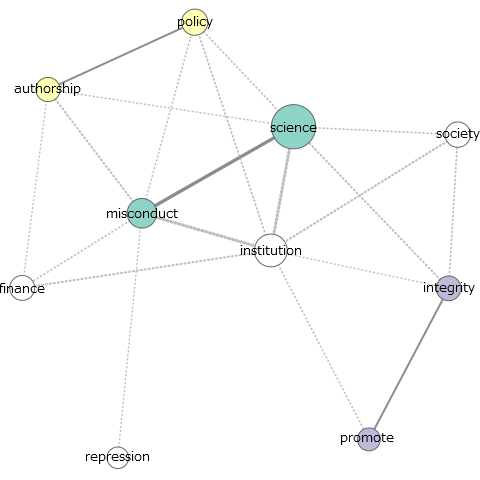


Figure C9 *Co-occurrence network of themes in OLD (1987-1990) newspaper articles*


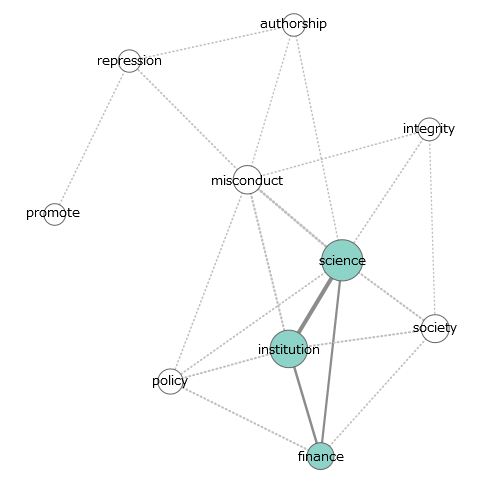


Figure C10 *Co-occurrence network of themes in MIDDLE (1995-2000) newspaper articles*


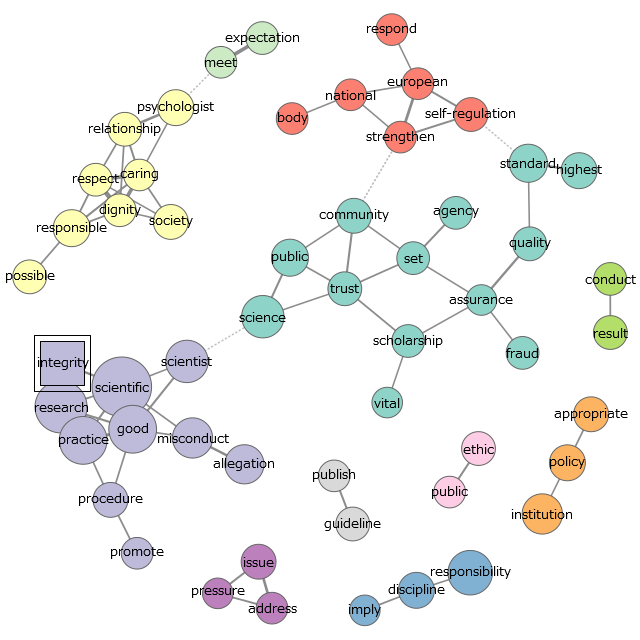


Figure C11 *Co-occurrence network of ‘integrity’ in OLD (1995-2001) policy documents*


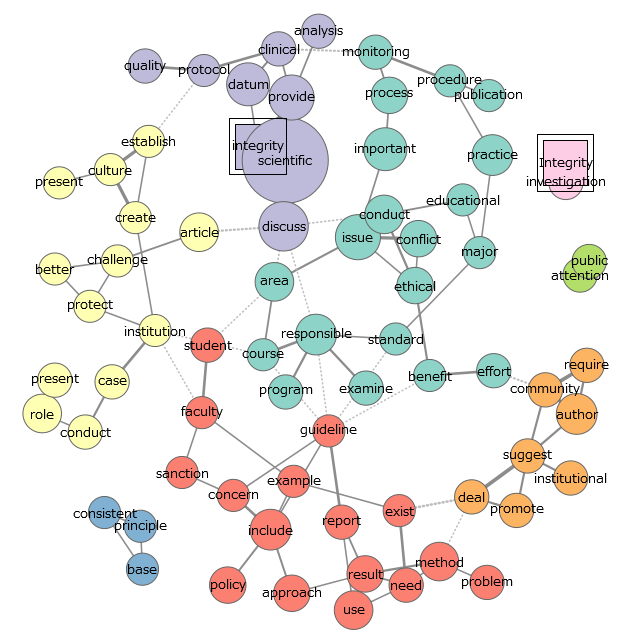


Figure C12 *Co-occurrence network of ‘integrity’ in the abstracts of 1991-1995 scientific publications*


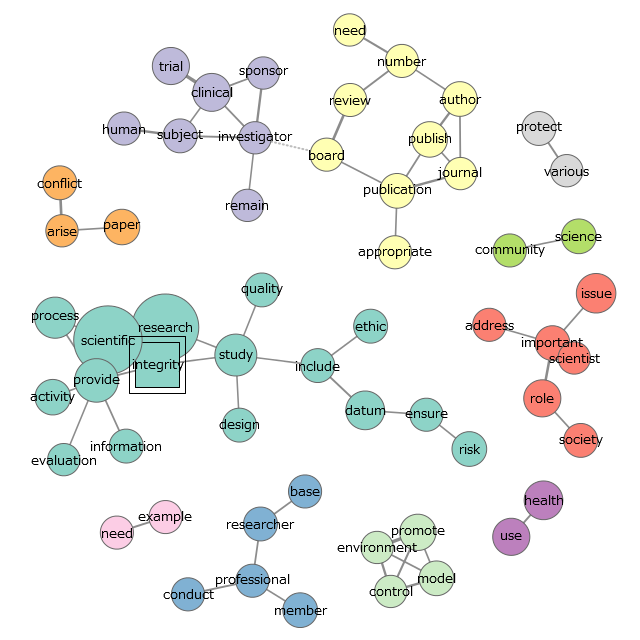


Figure C13 *Co-occurrence network of ‘integrity’ in the abstracts of 2001-2005 scientific publications*


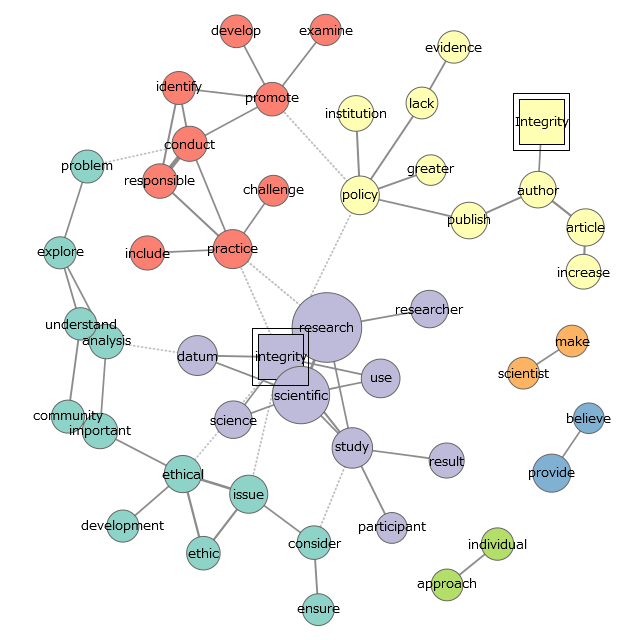


Figure C14 *Co-occurrence network of ‘integrity’ in the abstracts of 2011-2015 scientific publications*

Geographical comparison


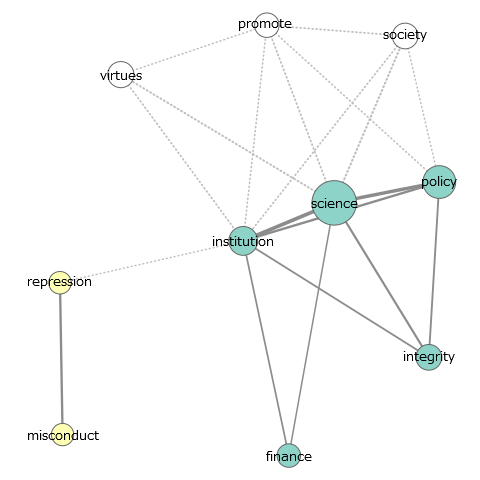


Figure C15 *Co-occurrence network of themes in British policy documents*


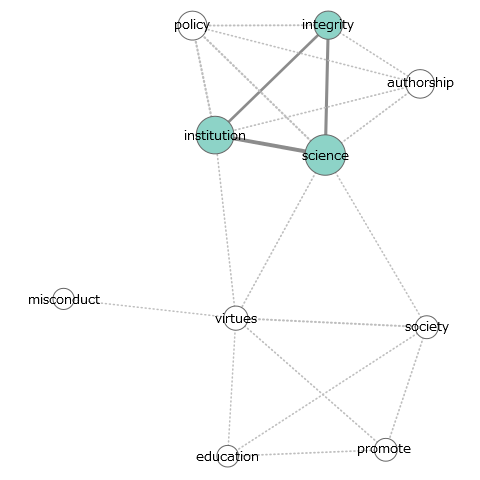

Figure C16 Co-occurrence network of themes in Dutch policy documents


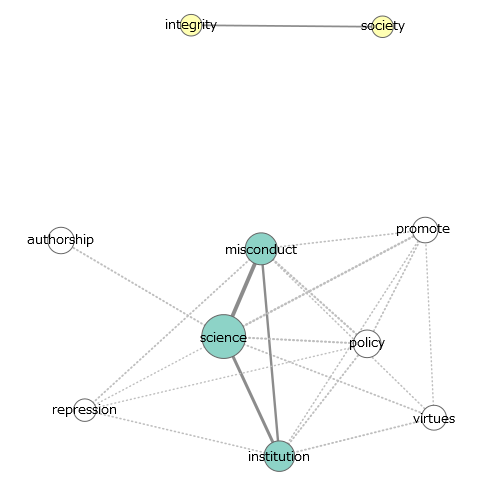


Figure C17 *Co-occurrence network of themes in German policy documents*


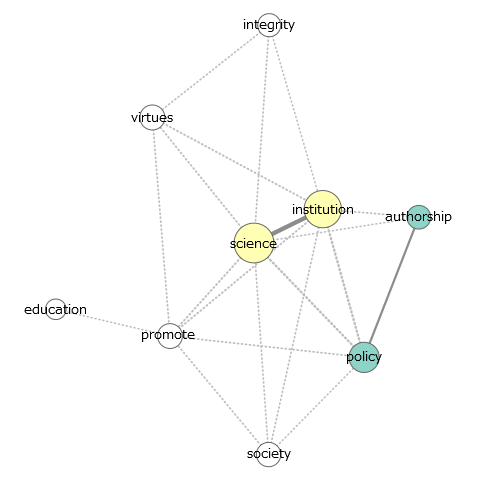


Figure C18 *Co-occurrence network of themes in Italian policy documents*


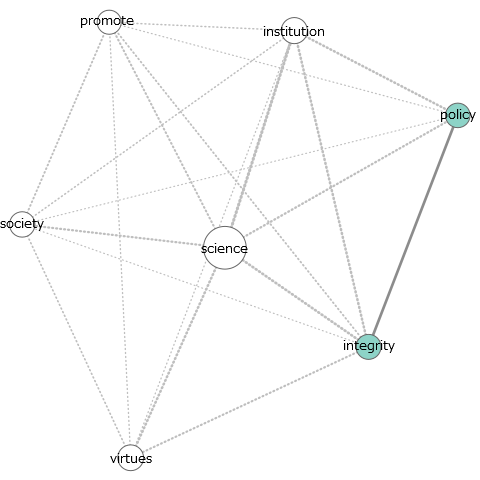


Figure C19 *Co-occurrence network of themes in Norwegian policy documents*


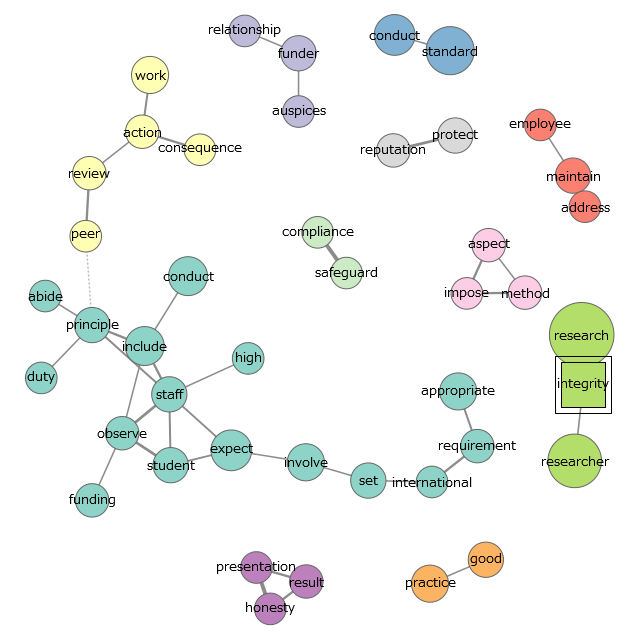


Figure C20 *Co-occurrence network of ‘integrity’ in British policy documents*


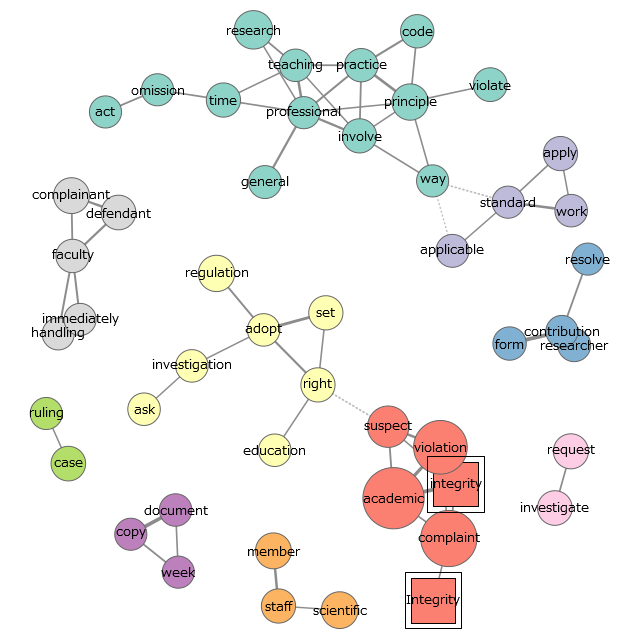


Figure C21 *Co-occurrence network of ‘integrity’ in Dutch policy documents*


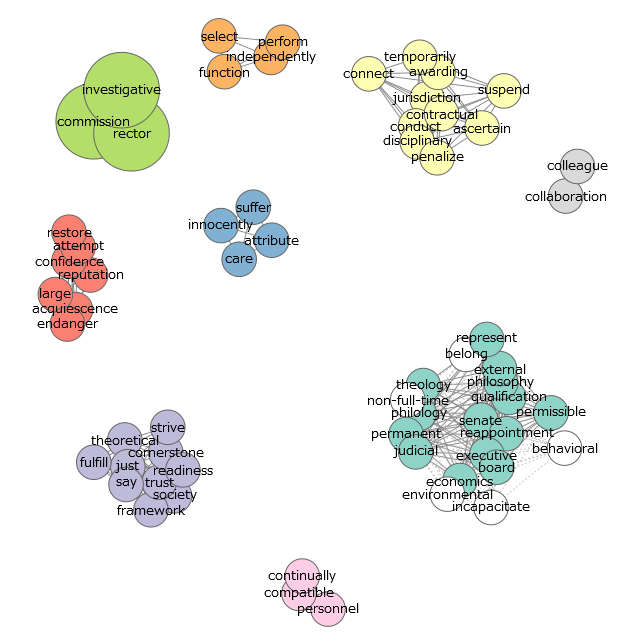


Figure C22 *Co-occurrence network of ‘integrity’ in German policy documents*


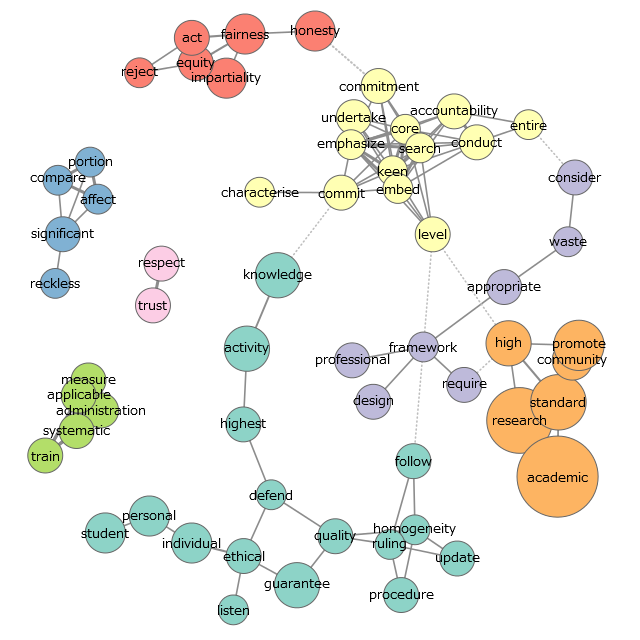


Figure C23 *Co-occurrence network of ‘integrity’ in Italian policy documents*


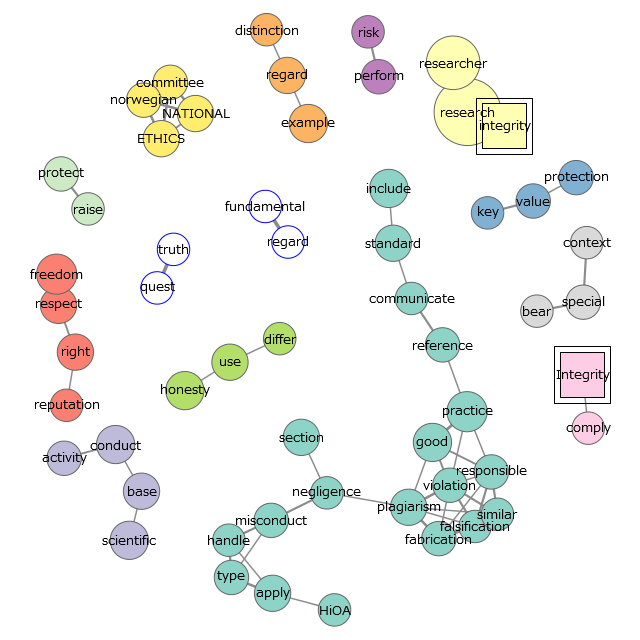


Figure C24 *Co-occurrence network of ‘integrity’ in Norwegian policy documents*
